# The Clinical Significance and Risk Factors of Anti-Platelet Factor 4/heparin Antibody on Maintenance Hemodialysis Patients: A Two-Year Prospective Follow-up

**DOI:** 10.1371/journal.pone.0062239

**Published:** 2013-04-30

**Authors:** Delong Zhao, Xuefeng Sun, Li Yao, Hongli Lin, Jijun Li, Jiuyang Zhao, Zhimin Zhang, Lide Lun, Jianrong Zhang, Mingxu Li, Qi Huang, Yang Yang, Shimin Jiang, Yong Wang, Hanyu Zhu, Xiangmei Chen

**Affiliations:** 1 Department of Nephrology, Chinese PLA General Hospital, State Key Laboratory of Kidney Disease (2011DAV00088), Beijing, China; 2 Department of Nephrology, The First Affiliated Hospital, Chinese Medical University, Shenyang, China; 3 Department of Nephrology, The First Affiliated Hospital, Dalian Medical College, Dalian, China; 4 Department of Nephrology, The First Affiliated Hospital, General Hospital of PLA, Beijing, China; 5 Department of Nephrology, The Second Affiliated Hospital, Dalian Medical College, Dalian, China; 6 Department of Nephrology, General Hospital of the General Headquarters, Beijing, China; 7 Department of Nephrology, General Hospital of the Air Force, Beijing, China; 8 Department of Nephrology, General Hospital of the Force Police Army, Beijing, China; 9 Department of Nephrology, General Hospital of the Navy, Beijing, China; General Hospital of the General Headquarters, China

## Abstract

**Background:**

Heparin-induced thrombocytopenia is an immune response mediated by anti-PF4/heparin antibody, which is clinically characterized by thrombocytopenia and thromboembolic events. In this study, a prospective and multi-center clinical investigation 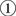 determined the positive rate of anti-PF4/heparin antibody in maintenance hemodialysis patients in China, 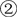 identified the related risk factors, and 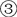 further explored the effect of the anti-PF4/heparin antibody on bleeding, thromboembolic events, and risk of death in the patients.

**Methods:**

The serum anti-PF4/heparin antibody was measured in 661 patients from nine hemodialysis centers, detected by IgG-specific ELISA and followed by confirmation with excess heparin. Risk factors of these patients were analyzed. Based on a two-year follow-up, the association between the anti-PF4/heparin antibody and bleeding, thromboembolic events, and risk of death in the patients was investigated.

**Results:**

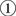
 The positivity rate of the anti-PF4/heparin antibody in maintenance hemodialysis patients was 5.6%. With diabetes as an independent risk factor, the positivity rate of the anti-PF4/heparin antibody decreased in the patients undergoing weekly dialyses ≥3 times. 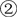 The positivity rate of the anti-PF4/heparin antibody was not related to the occurrence of clinical thromboembolic events and was not a risk factor for death within two years in maintenance hemodialysis patients. 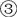 Negativity for the anti-PF4/heparin antibody combined with a reduction of the platelet count or combined with the administration of antiplatelet drugs yielded a significant increase in bleeding events. However, the composite determination of the anti-PF4/heparin antibody and thrombocytopenia, as well as the administration of antiplatelet drugs, was not predictive for the risk of thromboembolic events in the maintenance hemodialysis patients.

**Conclusions:**

A single detection of the anti-PF4/heparin antibody did not predict the occurrence of clinical bleeding, thromboembolic events, or risk of death in the maintenance hemodialysis patients.

## Introduction

Hemodialysis is currently the major treatment method for end-stage renal disease (ESRD). Hemodialysis is a treatment model of extracorporeal circulation, and the heparin anticoagulants are its main anticoagulant drugs [1]. Heparin-induced thrombocytopenia (HIT) is one of the serious adverse effects of heparin, which often results in severe thrombotic diseases [2], [3]. The pathogenesis of HIT mainly involves the binding of heparin to platelet factor 4 (PF4) to form a heparin-PF4 complex that stimulates the body to produce anti-PF4/heparin antibodies and mediates an immune response, which leads to platelet activation and reduction and results in an elevated risk of thromboembolic disease [4]–[6].

Due to the long-term administration of heparin, hemodialysis patients have a high risk [7]of positivity for the anti-PF4/heparin antibody with a reported positivity rate of 1.2% – 10.3% for the anti-PF4/heparin antibody [8]–[12]; by contrast, other researchers determined that the positivity rate of the anti-PF4/heparin antibody is as high as 47% [13]. Several studies suggested that the anti-PF4/heparin antibody increases the occurrence of thrombotic events in maintenance hemodialysis (MHD) patients [14], [15], but these results were different. The main reason for the variations in the results from most of the investigations was the use of a single center and a small sample size, which was not sufficient to rule out the variations caused by the different hemodialysis centers. The positivity rate of the anti-PF4/heparin antibody in MHD patients from a large sample and from multi-center resources in China is currently not available. Thus, in the present study, we prospectively examined the anti-PF4/heparin antibody in 661 MHD patients from nine hemodialysis centers using a two-year follow-up period to ? determine the positivity rate of the anti-PF4/heparin antibody in the Chinese MHD patients; ? resolve its related risk factors; and ? explore the effect of the anti-PF4/heparin antibody on the occurrence of bleeding, thromboembolic events, and the risk of death in the MHD patients.

## Methods

### Recruitment of the patients and healthy controls

**Figure 1:**
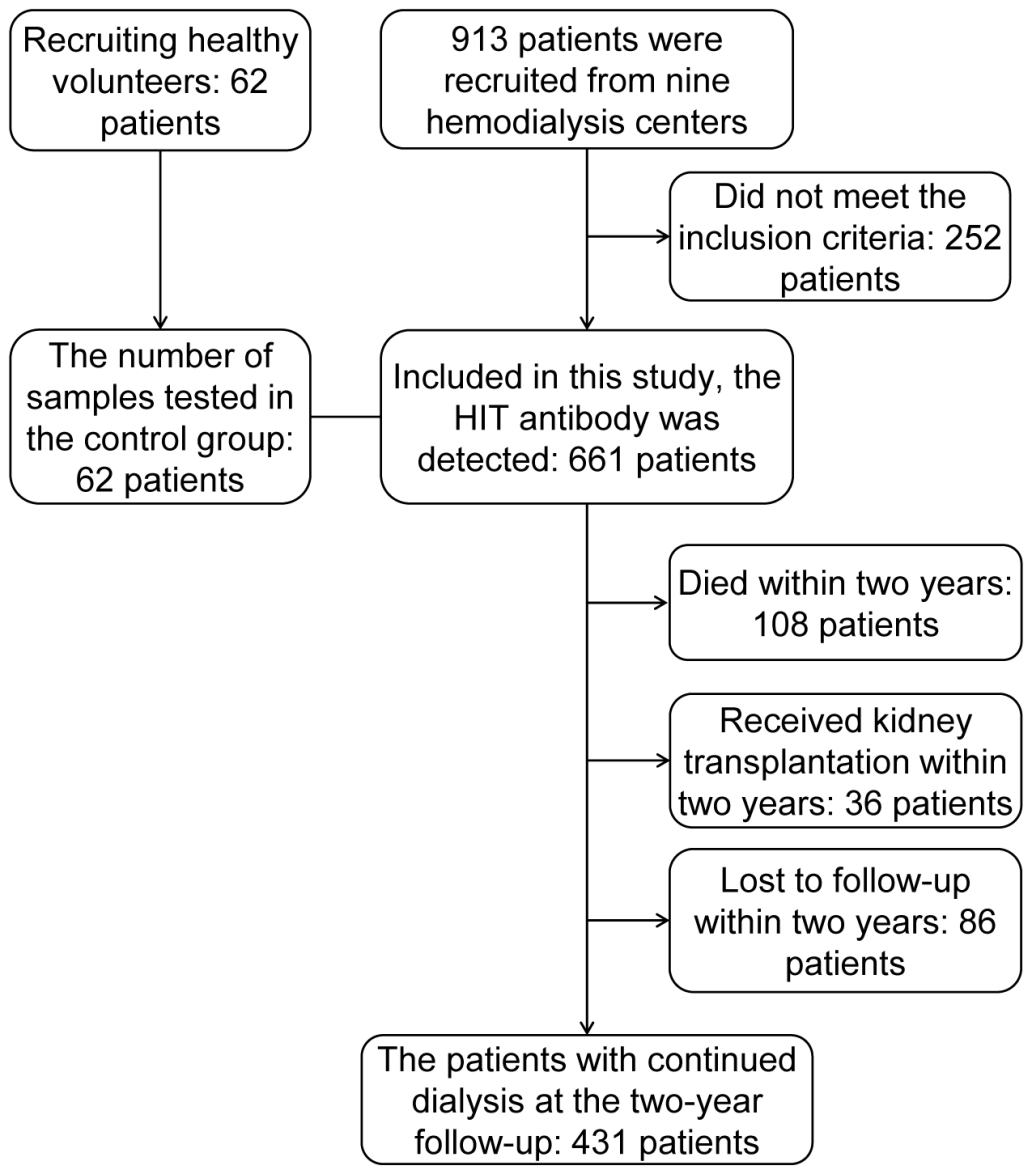
Diagram of MHD patients and healthy controls. The diagram shows the numbers of MHD patients and healthy controls who met the criteria of inclusion in or exclusion from the study and the follow-up of the MHD patients.

This study was approved by the Ethics Committee of the Chinese People’s Liberation Army (PLA) General Hospital and involved the hemodialysis centers of nine hospitals in three cities in northern China; 913 patients were recruited from December 2009 to January 2010. The patient inclusion criteria included the following: (1) maintenance hemodialysis for three months or longer; (2) age greater than 14 years, whether male or female; (3) use of heparin or low-molecular-weight heparin (LMWH) as an anticoagulant; and (4) provision of signed, informed consent. Consequently, 661 patients fulfilled these criteria and were included in this study. (Figure 1)

Concurrently, 62 healthy subjects with ages ranging from 18 – 80 years were selected as the control group. Based on the standardized definition of health from on the SENIEUR protocol [16], the subjects who met all of the following criteria were included in the study: (1) serum liver enzyme level (both serum glutamate oxaloacetate transaminase, GOT, and serum glutamate pyruvate transaminase, GPT) <40 U/L, fasting blood glucose (FBG) <7.1 mmol/L; serum urea nitrogen (UN) 1.8 – 7.5 mmol/L, and serum creatinine (SCr) 50 – 133 µmol/L; (2) hemoglobin (Hb) 110 – 176 g/L, hematocrit (HCT) 0.40 – 0.52 for men and 0.37 – 0.47 for women; (3) normal routine urine test results; (4) absence of any clinical history of visceral (including heart, brain, lung, kidney, and liver) disease, malignancy, rheumatic disease, and chronic infectious disease; (5) no history of exposure to heparin drugs; (6) normal electrocardiogram, chest radiograph, echocardiography, and abdominal ultrasound; and (7) sound character, stable emotions, absence of mental disorders, teachability, and ability to adapt to the environment and to engage in appropriate social interactions and interpersonal relationships.

The heparin group was defined as patients who used heparin as a hemodialysis anticoagulant or to pre-rinse the dialyzer and had used heparin as a long-term central venous catheter anticoagulant. The LMWH group was defined as patients who used LMWH as the hemodialysis anticoagulant and had never been exposed to heparin.

### Collecting the clinical data from the research subjects

The clinical data from the MHD patients were collected by the physicians in each hemodialysis center. The recorded contents included general information (i.e., age, sex, and dry weight), general clinical information (i.e., primary disease of renal failure, drug application, bleeding, and thromboembolic adverse events within the past three months), dialysis-related information (i.e., the dialysis duration in months, the number of weekly dialyses, and the type and amount of anticoagulant), and related laboratory tests (i.e., platelet count, routine blood test, routine urine test, and biochemical indicators). For the healthy controls, data regarding age, sex, weight, blood pressure, and laboratory indicators were determined and recorded. Written informed consent was obtained from each participant. Volunteers who did not meet the inclusion criteria or did not sign the informed consent were excluded from the study.

### Definition of previous bleeding and thromboembolic events

Within three months prior to the collection of the clinical information, the clinical bleeding events included conjunctival hemorrhage, bleeding due to the puncture needle, gastrointestinal bleeding, skin ecchymosis, and cerebral hemorrhage. The thromboembolic events included peripheral venous thrombosis, peripheral arterial thrombosis, vascular access occlusion, pulmonary embolism, cerebral infarction, and myocardial infarction. All records of bleeding and thromboembolic events were based on the medical records, laboratory tests, and imaging studies.

### Follow-up of the MHD patients

Two years after the detection of the anti-PF4/heparin antibody, follow-up was performed in the 661 patients in the study, of which 431 patients (65.20%) continued dialysis, 108 patients (16.34%) died, 36 patients (5.45%) received renal transplantation within two years, and 86 patients (13.01%) were lost to follow-up because they decided to discontinue the hemodialysis or their addresses were changed due to relocation. All of the above mentioned data were collected again from the 431 patients with continued dialysis and a complete two-year follow-up. The bleeding and/or thromboembolic events that occurred between two years from the first data collection and the time of follow-up were also recorded. For the patients who died within two years, the reasons for their bleeding, thromboembolic events, and deaths were ascertained from the medical records.

### Collection of blood samples and detection of the serum anti-PF4/heparin antibody

For the 661 MHD patients included in this study, the predialysis blood samples were collected at various hemodialysis centers between December 2009 and January 2010, and the sera were isolated and transported in aliquots to the State Key Laboratory of Kidney Disease. The State Key Laboratory of Kidney Disease selected the volunteers for the healthy control group, collected their blood samples, and isolated and stored the sera in aliquots. All of the serum samples were stored at –80°C in the State Key Laboratory of Kidney Disease until the unified testing.

The serum anti-PF4/heparin antibody was detected using a heparin/PF4 ELISA kit (PF4 IgG™, HAT45G, GTI Diagnostics, Waukesha, WI, USA) [17]. The unified testing was conducted by certain technical staff members in strict accordance with the kit’s instruction guide. The positive control yielded OD405 nm >1.8, and the negative control yielded OD405 nm <0.3 with a difference between the duplicate wells of <20% [18]. To avoid false positives and improve the detection specificity [19], [20], the high-dose heparin confirmatory tests were performed for all of the samples with OD405 nm >0.4. The samples with OD405 nm >0.4 and an inhibition test result >50% were defined as positive for the anti-PF4/heparin antibody.

### Data analysis

The chi-square test was used to compare the categorical variables. The t-test or Mann-Whitney U test was used for the continuous variables. Multivariate logistic regression was applied to analyze the correlation between positivity for the anti-PF4/heparin antibody and the risk factors of death in the MHD patients, as well as the correlation between positivity for the anti-PF4/heparin antibody and the occurrence of bleeding and thromboembolic events within two years. P value <0.05 was considered statistically significant. The statistical software SPSS version 17.0 was used in this study.

## Results

### General clinical data from the research subjects

In total, 62 healthy controls and 661 MHD patients met the inclusion criteria for this study, and their clinical data are presented in Table 1.

**Table 1:** MHD patients and healthy controls characteristics according to baseline status.

	MHD patients		healthy controls	
Sex (female;n [%])	287	(43%)	39	(63%)
Age (years;median [25th to 75th percentiles])	57	(44–70)	60	(55–69)
Dry Weight(kg;median [25th to 75th percentiles])	62	(54–71)	65	(55–70)
BMI (mean [SD])	22.6±4.0		24.3±3.0	
SBP(mmHg;median [25^th^ to 75^th^ percentiles])	140	(130–155)	130	(120–135)
DBP(mmHg;median [25^th^ to 75^th^ percentiles])	80	(70–90)	78	(70–80)
Hb(g/L; median [25^th^ to 75^th^ percentiles])	105	(94–115)	143	(135–147)
Platelet count (10?9/L; mean [SD])	167±58		222±53	
Thrombocytopenia (n [%])	80	(12%)	0	(0%)
SCr(µmol/L; mean [SD])	883±313		67±12	
ALB(g/L;median [25th to 75th percentiles])	40	(37–42)	46	(44–48)
Diabetes(n [%])	127	(19%)	0	(0%)
Bleeding events within the past three months (n [%])	83	(13%)	0	(0%)
Thromboembolic events within the past three months (n [%])	66	(10%)	0	(0%)
Dialysis duration (months ;n [%])				
3∼6	73	(11%)	–	
7∼12	69	(10%)	–	
13∼36	205	(31%)	–	
37∼60	149	(23%)	–	
> = 60	165	(25%)	–	
Number of weekly dialyses (n [%])				
<3	209	(32%)	–	
> = 3	452	(68%)	–	
Dialysis membrane (n [%])				
Non-synthetic membrane	71	(11%)	–	
synthetic membrane	590	(89%)	–	
Dialyzer (n [%])				
Low-flux	307	(46%)	–	
Middle-flux	238	(36%)	–	
High-flux	116	(18%)	–	
Anticoagulant (n [%])				
heparin	578	(87%)	–	
LMWH	83	(13%)	–	
Kt/v(;median [25th to 75th percentiles])	1.421	(1.245–1.619)	–	

BMI: body mass index; SBP: systolic blood pressure; DBP: diastolic blood pressure; Hb:hemoglobin; SCr: serum creatinine; ALB: albumin.

### The positivity rate of the serum anti-PF4/heparin antibody and its risk factors in the research subjects

The detection of the serum anti-PF4/heparin antibody in the 62 healthy subjects was negative. Among the 661 MHD patients, 37 subjects tested positive for the anti-PF4/heparin antibody (positivity rate of 5.6%), of which 33 individuals had a history of heparin exposure, and the remaining four cases had a history of exposure to LMWH only. Moreover, the 37 patients who tested positive for the anti-PF4/heparin antibody had no clinical diagnosis of HIT. The positivity rate of the anti-PF4/heparin antibody in the MHD patients did not significantly differ among the nine hemodialysis centers. The univariate analysis revealed that the positivity rate of the serum anti-PF4/heparin antibody was significantly increased in the MHD patients whose primary diseases were diabetes and elevated diastolic blood pressures (DBPs). No significant difference between the groups testing positive versus negative for the serum anti-PF4/heparin antibody was found in age, sex, dry weight, body mass index (BMI), systolic blood pressure (SBP), hemoglobin, platelet count and thrombocytopenia (defined as PLT <100×10^9^/L), serum creatinine, albumin, bleeding events within the past three months, thromboembolic events within the past three months, dialysis duration in months, number of weekly dialyses, dialysis membrane, dialyzer, anticoagulant, and Kt/v (Table 2).

**Table 2:** Results of univariate analysis.

	Anti-PF4/heparin antibody							
	Positive (n = 37)			Negative (n = 624)			*P* value	
Age(years;mean [SD])	56±17			56±16			0.944	
Sex (female;n [%])	15	(41%)		272	(44%)	0.737		
Dry Weight(kg;median [25th to 75th percentiles])	61.5	(54–71)		62.8	(55–70)	0.706		
BMI (mean [SD])	23.4±5.5			22.6±3.9		0.236		
SBP(mmHg; mean [SD])	148±19			141±21			0.066	
DBP(mmHg; mean [SD])	86±13			80±13			0.032	
Hb(g/L; median [25^th^ to 75^th^ percentiles])	105		(94–115)	105	(90–118)		0.904	
Platelet count (10?9/L; mean [SD])	166±58			178±53			0.144	
Thrombocytopenia (n [%])	4		(11%)	76	(12%)		0.804	
SCr(µmol/L; mean [SD])	885±314			838±279			0.493	
ALB(g/L;median [25th to 75th percentiles])	40		(37–42)	40	(38–42)		0.811	
Diabetes(n [%])	13		(35%)	114	(18%)		0.017	
Bleeding events within the past three months (n [%])	4		(11%)	79	(13%)		0.941	
Thromboembolic events within the past three months (n [%])	3		(8%)	63	(10%)		0.913	
Dialysis duration (months ;n [%])								
3∼6	7		(19%)	66	(11%)		0.305	
7∼12	6		(16%)	63	(10%)			
13∼36	11		(30%)	194	(31%)			
37∼60	6		(16%)	143	(23%)			
> = 60	7		(19%)	158	(25%)			
Number of weekly dialyses (> = 3 times; n [%])	20		(54%)	432	(69%)		0.095	
Dialysis membrane (synthetic membrane; n [%])	32		(86%)	558	(89%)		0.582	
Dialyzer (n [%])								
Low-flux	19		(51%)	288	(46%)		0.855	
Middle-flux	12		(33%)	226	(36%)			
High-flux	6		(16%)	110	(18%)			
Anticoagulant (heparin; n [%])	33		(89%)	545	(87%)		0.809	
Kt/v(;median [25th to 75th percentiles])	1.295		(1.097–1.492)	1.430	(1.250–1.620)		0.104	

BMI: body mass index; SBP: systolic blood pressure; DBP: diastolic blood pressure; Hb:hemoglobin; SCr: serum creatinine; ALB: albumin.

The multivariate logistic regression analysis revealed that diabetes (OR  = 4.405, 95% CI [1.573 – 12.334], *P* = 0.005) significantly increased the positivity rate of the serum anti-PF4/heparin antibody in the MHD patients, and the number of weekly dialyses (≥3 times) reduced the positivity rate of the serum anti-PF4/heparin antibody (OR  = 0.324, 95% CI [0.114 – 0.925], *P* = 0.035). The positivity rate of the serum anti-PF4/heparin antibody was not significantly correlated with age, sex, body weight, BMI, SBP, DBP, hemoglobin, platelet count and thrombocytopenia, serum creatinine, albumin, bleeding events within the past three months, thromboembolic events within the past three months, the dialysis duration in months, the number of weekly dialyses, dialysis membrane, dialyzer, anticoagulant, and Kt/v (Table 3).

**Table 3:** Results of multivariate analysis.

	OR	95% C.I.	P value
Age	1.002	0.969–1.037	0.888
Sex (female])	0.877	0.411–1.870	0.733
Dry Weight	0.968	0.926–1.011	0.144
BMI	0.992	0.867–1.136	0.911
SBP	0.999	0.973–1.026	0.948
DBP	1.041	0.993–1.092	0.094
Hb	1.006	0.980–1.033	0.640
Platelet count	1.000	0.990–1.011	0.971
Thrombocytopenia	0.796	0.116–5.463	0.817
SCr	1.000	0.998–1.001	0.626
ALB	1.030	0.946–1.122	0.498
Diabetes	4.405	1.573–12.334	0.005
Bleeding events within the past three months	0.535	0.062–4.576	0.568
Thromboembolic events within the past three months	0.838	0.167–4.206	0.830
Dialysis duration (months)			
7∼12	0.509	0.085–3.049	0.460
13∼36	0.452	0.111–1.850	0.270
37∼60	0.478	0.100–2.293	0.356
> = 60	0.671	0.139–3.233	0.619
Number of weekly dialyses (> = 3 times)	0.324	0.114–0.925	0.035
Dialysis membrane (synthetic membrane)	1.204	0.088–16.418	0.889
Anticoagulant (heparin)	0.654	0.188–2.281	0.505
Kt/v	0.283	0.041–1.931	0.197

OR: odds ratio; CI: confidence interval; BMI: body mass index; SBP: systolic blood pressure; DBP: diastolic blood pressures; Hb:hemoglobin; SCr: serum creatinine; ALB: albumin.

### The risk factors for death in the MHD patients

At the two-year follow-up, among the 661 MHD patients, there were 431 cases with continued dialysis, 108 cases of death, 36 cases of kidney transplant or peritoneal dialysis, and 86 cases lost to follow-up. The 37 patients who tested positive for the anti-PF4/heparin antibody included 21 cases with continued dialysis, six cases of death, two cases of kidney transplantation or peritoneal dialysis, and eight cases lost to follow-up. Among the 539 patients with complete follow-up data (431 cases with continued dialysis and 108 cases of death), the two-year cumulative mortality rate for the MHD patients was 20.0% with an average annual mortality rate of 10.6%. The multivariate logistic regression analysis revealed that advanced age and diabetes mellitus significantly increased the risk of death in the patients undergoing maintenance hemodialysis, whereas utilizing a synthetic membrane dialyzer significantly reduced the patient's risk of death. The death of the patients was not significantly associated with the sex, body weight, SBP, DBP, anti-PF4/heparin antibody, hemoglobin, platelet count and thrombocytopenia, serum creatinine, albumin, bleeding events within the past three months, thromboembolic events within the past three months, dialysis duration in months, number of weekly dialyses (≥3), dialyzer, anticoagulant, and Kt/v (*P*>0.05). (Table 4)

**Table 4:** The baseline-factor analysis between the patients with continued dialysis and death.

	Continued dialysis (n = 431)		Death (n = 108)		OR	95% C.I.	*P* value
Age (years;median [25th to 75th percentiles])	55	(27–77)	67	(43–85)	1.069	(1.039–1.099)	<0.001
Sex (female;n [%])	201	(47%)	45	(42%)	0.564	(0.292–1.089)	0.088
Dry Weight	60.8	(54–68)	61.1	(55–69)	1.002	(0.971–1.033)	0.922
BMI	22.5±3.8		22.5±4.0		1.047	(0.968–1.131)	0.250
SBP(mmHg; mean [SD])	141±21		142±22		1.003	(0.988–1.019)	0.681
DBP(mmHg; mean [SD])	81±12		76±14		1.002	(0.973–1.032)	0.880
Anti-PF4/heparin antibody(positive;n [%])	21	(5%)	6	(6%)	1.267	(0.336–4.780)	0.727
Hb(g/L; median [25^th^ to 75^th^ percentiles])	107	(96–116)	102	(91–115)	0.994	(0.977–1.011)	0.506
PLT(10?9/L; mean [SD])	167±57		163±59		0.998	(0.992–1.005)	0.607
thrombocytopenia (n [%])	47	(11%)	13	(12%)	0.451	(0.139–1.464)	0.185
SCr(µmol/L; mean [SD])	908	(344–1392)	705	(208–1301)	1.000	(0.999–1.001)	0.750
ALB(g/L;median [25th to 75th percentiles])	40	(32–45)	37	(29–45)	0.966	(0.911–1.025)	0.253
diabetes(n [%])	68	(16%)	41	(38%)	2.061	(1.062–4.000)	0.033
Bleeding events within the past three months (n [%])	60	(14%)	14	(13%)	0.963	(0.396–2.340)	0.934
Thromboembolic events within the past three months (n [%])	39	(9%)	17	(16%)	1.624	(0.724–3.641)	0.239
Time on dialysis (months ;n [%])							
3∼6	27	(6%)	13	(12%)	1.000	–	–
7∼12	50	(12%)	10	(9%)	0.835	(0.189–3.683)	0.812
13∼36	127	(29%)	41	(38%)	2.118	(0.617–7.271)	0.233
37∼60	104	(24%)	25	(23%)	1.321	(0.365–4.779)	0.671
> = 60	123	(29%)	19	(18%)	1.175	(0.309–4.472)	0.813
Number of weekly dialyses (> = 3 times; n [%])	281	(65%)	85	(79%)	1.792	(0.805–3.991)	0.153
Dialysis membrane (synthetic membrane;n [%])	388	(90%)	96	(89%)	0.145	(0.024–0.885)	0.036
Dialyzer (n [%])							
Low-flux	199	(46%)	59	(55%)	1.000	–	–
Middle-flux	158	(37%)	32	(30%)	0.879	(0.400–1.935)	0.749
High-flux	74	(17%)	17	(16%)	1.130	(0.453–2.818)	0.794
Anticoagulant (heparin; n [%])	381	(88%)	88	(81%)	0.693	(0.323–1.485)	0.345
Kt/v(;median [25th to 75th percentiles])	1.430	(1.266–1.640)	1.462	(1.230–1.599)	0.681	(0.285–1.623)	0.386

OR: odds ratio; CI: confidence interval; BMI: body mass index; SBP: systolic blood pressure; DBP: diastolic blood pressures; Hb:hemoglobin; SCr: serum creatinine; ALB: albumin.

### Prediction of bleeding and thromboembolic events in the MHD patients based on the serum anti-PF4/heparin antibody combined with either thrombocytopenia or the administration of antiplatelet drugs

Of the 431 patients with continued hemodialysis after two years of follow-up, 159 patients experienced new bleeding events within the two years with an incidence of 36.9% for the two-year cumulative bleeding events and an average annual incidence of 20.6%; 91 patients experienced new thromboembolic events with an incidence of 21.1% for the two-year cumulative thromboembolic events and an average annual incidence of 11.2%. According to the baselines of the anti-PF4/heparin antibody and platelet count or the anti-PF4/heparin antibody and antiplatelet drugs, the patients with continued MHD after two years of follow-up were divided into four groups: antibody negative + normal platelets, antibody negative + reduced platelets, antibody positive + normal platelets, and antibody positive + reduced platelets. In addition, there were four groups, as follows: antibody negative without antiplatelet drug administration, antibody negative with antiplatelet drug administration, antibody positive without antiplatelet drug administration, and antibody positive with antiplatelet drug administration. Respectively taking the group of antibody negative + normal platelets or the group of antibody negative without antiplatelet drug administration as the standard group (the OR value was set to 1), the risk of the occurrence of bleeding events and thromboembolic events during the two-year follow-up was analyzed for the patients in the other groups. The results revealed that the group of antibody negative + reduced platelets (OR  = 1.890, 95% CI [1.014 – 3.522], *P* = 0.045) and the group of antibody negative with antiplatelet drug administration (OR  = 1.586, 95% CI [1.034 – 2.432], *P* = 0.034) had a significantly increased risk of occurrence of bleeding events. The risks of bleeding events in the other groups were not significantly different from those in the standard groups. No significant difference was found in the risk of clinical thromboembolic events between the groups (Table 5).

**Table 5:** Analysis of clinical bleeding & thromboembolic events within the two years.

	Bleeding events within the two years					Thromboembolic events within the two years				
	n/n, (percentage)		OR	95% C.I.	*P* value	n/n, (percentage)		OR	95% C.I.	*P* value
antibody negative + normal platelets	130/365	(36%)	1.000	—	0.155	71/365	(19%)	1.000	—	0.220
antibody negative + reduced platelets	23/45	(51%)	1.890	1.014–3.522	0.045	13/45	(29%)	1.682	0.840–3.370	0.142
antibody positive + normal platelets	5/20	(25%)	0.603	0.214–1.696	0.337	7/20	(35%)	2.230	0.858–5.792	0.100
antibody negative + reduced platelets	1/1	(100%)	—	—	>0.999	0/1	(0%)	—	—	>0.999
antibody negative without antiplatelet drug administration	96/283	(34%)	1.000	—	0.153	59/283	(21%)	1.000	—	0.401
antibody negative with antiplatelet drug administration	57/127	(45%)	1.586	1.034–2.432	0.034	25/127	(20%)	0.931	0.552–1.570	0.787
antibody positive without antiplatelet drug administration	3/12	(25%)	0.649	0.172–2.454	0.524	3/12	(25%)	1.266	0.332–4.822	0.730
antibody positive with antiplatelet drug administration	3/9	(33%)	0.974	0.238–3.980	0.971	4/9	(44%)	3.037	0.791–11.666	0.106

Values are shown as the Number of bleeding (or thromboembolic) events/the number of the patients of the group, (percentage). OR: odds ratio; CI: confidence interval.

## Discussion

In this study, we first investigated the serum anti-PF4/heparin antibody levels in 661 MHD patients from nine hemodialysis centers in three cities of northern China, in which 37 patients tested positive for the anti-PF4/heparin antibody, with a positivity rate of 5.6% being observed for the serum anti-PF4/heparin antibody. According to the diagnosis criteria[6], clinicians make a diagnosis of HIT when any of the following events occurs in association with the presence of “HIT antibodies” detected by in vitro assays: (1) an unexplained platelet count fall; (2) venous or arterial thrombosis; (3) skin lesions at heparin injection sites; or (4) acute systemic (anaphylactoid) reactions that occur after IV heparin bolus administration. Although some of the patients tested positive for the antibody also had thrombocytopenia, bleeding or thromboembolic events within the past three months, not a single patient was clinically diagnosed with HIT. The main reason for the low diagnosis rate is that the detection of anti-PF4/heparin antibodies is not a routine clinical test in China. In addition, few publications regarding HIT-related research on hemodialysis patients are available. Therefore, it is necessary to improve the clinical detection of anti-PF4/heparin antibodies in China.

Nearly a decade of research on HIT has revealed positivity rates of the anti-PF4/heparin antibody that are mostly within the range of 1.2% to 10.3% [8]–[12]; however, it has also been reported that the positivity rate of the anti-PF4/heparin antibody in maintenance dialysis patients is as high as 17.9% [21], and even up to 47% [13]. One of the reasons for the difference between the results of the individual reports is that most of the studies were based on a single-center resource; thus, the results are not representative. A study of 305 MHD patients from four centers in Japan revealed a 2.3% positivity rate of the anti-PF4/heparin antibody [11]. The CHOICE Cohort Study in the USA showed that the positivity rate of the anti-PF4/heparin antibody was 10.6% among 596 selected MHD patients [10]. Our present study demonstrated a serum anti-PF4/heparin antibody positivity rate of 5.6% among 661 MHD patients from nine centers in China, indicating that the positivity rate of the anti-PF4/heparin antibody might differ in MHD patients of various nationalities and ethnicities. However, the positivity of HIT antibody is also largely influenced by the kit and detection method used in a study [19]. The kit and detection method used in the previous studies were varied, and this variety can not be excluded from the reasons for the difference of anti-PF4/heparin antibody positivity rate in these studies. Currently, the major method of detection of anti-PF4/heparin antibodies is ELISA, in which the sensitivity can be as high as 80% – 100%. However, the specificity is low because of the simultaneous detection of non-specific antibodies [22], [23]. Studies have shown that only the IgG class of anti-PF4/heparin antibodies are platelet-activating and thus have the potential to cause heparin-induced thrombocytopenia [19], [24]–[26]. Therefore, in this study, we only detected the IgG anti-PF4/heparin antibody. Additionally, to improve the specificity of detection of the anti-PF4/heparin antibody, the high-dose heparin confirmatory tests were performed [19], [20], [27] to set the positive standard of OD405 nm >0.4 and the inhibition test >50% for the anti-PF4/heparin antibody. This step ensured the reliability of the results in the present study, and the negative result for the serum anti-PF4/heparin antibody using the same detection method in the 62 healthy controls also confirmed the accuracy of the method applied in our study.

Moreover, the frequency of antibody formation depends on a variety of factors, including the chain length of the heparin molecule and degree of sulfation, amount and frequency of heparin administration, clinical setting, and degree of nonimmune platelet activation [28]. The results of this study indicates that 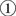 the positivity rate of the anti-PF4/heparin antibody in MHD patients is not associated with their age, sex, and dialysis duration, which is similar to previous findings [13], [21]. 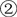 The positivity rate of the anti-PF4/heparin antibody is unrelated to the occurrence of thrombocytopenia, which is also consistent with previously reported results [21]. 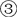 The positivity rate of the anti-PF4/heparin antibody in MHD patients undergoing three or more dialyses per week was significantly lower than that in patients undergoing less than three dialyses per week. Although a high frequency of dialyses could lead to an increased dose and frequency of heparin use (as well as increase the levels of heparin exposure in the patients), whether long-term and high-frequency exposure to heparin can induce immune tolerance in patients remains to be studied. 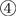 Although the diabetic MHD patients in this study were significantly more likely than the non-diabetic patients to undergo more than three dialyses per week (97/127 cases, 76.4% *vs* 355/530 cases, 67.0%, respectively; *P* = 0.043), the positivity rate of the anti-PF4/heparin antibody was significantly increased in the diabetic patients with MHD, which differs from the conclusion of Diaz J *et al.* [29] that diabetes does not increase the positivity rate of the anti-PF4/heparin antibody. The reason for the increased positivity rate of the anti-PF4/heparin antibody in the MHD patients remains unclear. 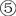 The result of this study revealed no significant difference in the positivity rate of the HIT antibody between the patients using heparin and LMWH. Although it is widely accepted that the frequency of HIT is greater in non-hemodialysis populations treated with unfractionated heparin compared with LMWH [30], it has also been reported that no significant difference in the positivity rate of the antibody in MHD patients was found between these two types of heparin[11].In this study, the patients in the heparin group included the patients using heparin as the hemodialysis anticoagulant or to pre-rinse the dialyzer, as well as the patients using heparin for long-term anticoagulation of the central venous catheter; furthermore, the patients who used LMWH as the hemodialysis anticoagulant and had never been exposed to heparin were defined as the LMWH group. Because the patients in the heparin group were significantly more numerous than the patients in the LMWH group (578/83 cases), the difference in the number of cases between the groups may reflect the difference in the positivity rate of the anti-PF4/heparin antibody between the two anticoagulants. 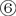 It was confirmed that the unified detection of the PF4/heparin antibody demonstrated no significant differences in the positivity rate of the anti-PF4/heparin antibody between the patients from the different hemodialysis centers in the same region of the same country. This finding might be related to the similar qualities and methods of applications of the heparin drugs used by hemodialysis patients in the same region of the same country.

The occurrence of HIT leads to platelet activation, thereby increasing the risk of occurrence of thromboembolic events in patients [31]–[34] and most likely increasing the mortality in MHD patients [9]. However, it has also been reported that an increase in anti-PF4/heparin antibody levels does not increase the incidence of thromboembolic events [8], [21] and the anti-PF4/heparin antibody had no effect on the overall mortality or cardiovascular events in the absence of thrombocytopenia[10]. Tsai YF et al. [34] reported that the anti-PF4/heparin antibody increases the risk of thromboembolic events, but they also determined that the anti-PF4/heparin antibody is not correlated with peripheral arterial disease (PAD) and coronary heart disease (CHD). In this study, a two-year follow-up was conducted for detection of the anti-PF4/heparin antibody in 661 MHD patients from nine centers, of which 431 patients (65.20%) were on continued dialysis and 108 patients (16.34%) died. The results further confirmed that the occurrence of thromboembolic events was not significantly associated with the anti-PF4/heparin antibody in the MHD patients, and testing positive for the anti-PF4/heparin antibody was not correlated with the two-year mortality of the patients. Further analysis of risk factors in the MHD patients who died revealed that advanced age and diabetes increased the risk of death, and dialysis with a synthetic membrane reduced the risk of death, which is consistent with findings of DOPPS [35]–[37]. However, no impact of the anti-PF4/heparin antibody on patient mortality was determined.

Based on the fundamental *in vitro* and *in vivo* experiments, antiplatelet drugs might prevent the occurrence of HIT [38], [39]; by contrast, several clinical trials have confirmed that antiplatelet drugs do not effectively suppress the occurrence of HIT [40], and antiplatelet drugs do not reduce the occurrence and degree of thrombocytopenia [41]. However, these studies were limited to case reports or research using a small sample size. Because the proportion of elderly patients and patients with diabetes and other cardiovascular complications (such as hypertension in MHD patients) has increased annually, the number of MHD patients taking antiplatelet drugs is also gradually increasing. In this study, the use of antiplatelet agents was an observational indicator, rather than an intervention. In the MHD patients who were using antiplatelet drugs, combined predictive effects were mainly observed for the presence of serum anti-PF4/heparin antibodies and the administration of antiplatelet drugs for bleeding and thromboembolic events. According to the baseline level of the anti-PF4/heparin antibody and antiplatelet drugs, the patients with continued MHD after the two years of follow-up were divided into four groups: antibody negative without antiplatelet drug administration, antibody negative with antiplatelet drug administration, antibody positive without antiplatelet drug administration, and antibody positive with antiplatelet drug administration. The results showed that the patients in the group of antibody negative with antiplatelet drug administration had a significantly increased risk for the occurrence of bleeding events during the two years of follow-up compared with the patients in the other three groups; whether the anti-PF4/heparin antibody was present and whether the patient was taking antiplatelet drugs had no significant effect on the occurrence of thromboembolic events during the two years of follow-up. In addition, the combined predictive effects of the serum anti-PF4/heparin antibody and the platelet count in MHD patients on the clinical bleeding and thromboembolic events were also observed. According to the baseline level of the anti-PF4/heparin antibody and platelet count, the patients with continued MHD after the two years of follow-up were divided into four groups: antibody negative + normal platelets, antibody negative + reduced platelets, antibody positive + normal platelets, and antibody positive + reduced platelets. The results showed that the patients in the antibody negative + reduced platelet group had a significantly increased incidence of bleeding events during the two-year follow-up compared with the patients in the other three groups, whereas neither the level of serum anti-PF4/heparin antibody nor the platelet count predicted the incidence of thromboembolic events. The pathogenesis of HIT by now are mainly regarded as HIT antibodies activate platelets intravascularly, causing the release of platelet microparticles and increased thrombin generation[42], [43]. Therefore, we consider that the activations of platelets by anti-PF4/heparin antibody, are likely to offset the risk of bleeding due to antiplatelet drugs and the decreased number of platelets. Although the majority of studies have reported an increased incidence of heparin-PF4 antibodies in patients with thromboembolic events, this has not been universal[8], [10], [13]. Therefore, we think that anti-PF4/heparin antibody or the activations of platelets are inadequate to cause thromboembolic events. Our data also showed that even considering the impact of the platelet count and antiplatelet drugs, the positive serum anti-PF4/heparin antibody can not predict the risk of occurrence of thromboembolic events in the MHD patients. In addition, the small number of patients tested positive for the serum anti-PF4/heparin antibody might potentially cause a deviation in the analysis.

The advantages of this study are as follows: 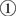 A standard detection method for the anti-PF4/heparin antibody was applied, which was verified by the healthy control. 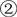 As a prospective and multi-center clinical cohort study with a large sample size, the present investigation should be more representative. 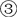 This study is the first multi-center investigation that addresses the anti-PF4/heparin antibody and its risk factors in Chinese MHD patients, at the level of clinical significance. The limitations of this study include the following: 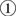 lack of dynamic monitoring and recording during the two years of follow-up (with the indicators being observed at multiple time points); 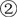 failure to redetect the serum anti-PF4/heparin antibody after the two years of observation; 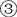 use of antiplatelet drugs as an observational indicator, rather than an intervention; 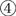 the smaller number of patients who tested positive for the serum anti-PF4/heparin antibody, which might have potentially caused a deviation in the analysis.

In summary, this study is the first to indicate that the positivity rate of the anti-PF4/heparin antibody in Chinese MHD patients was 5.6% using a prospective and multi-center clinical cohort study with a large sample size. We further verified that a single detection of anti-PF4/heparin antibody did not predict the occurrence of bleeding, thromboembolic events, and risk of death in the MHD patients. Finally, we demonstrated that the patients who tested negative for the anti-PF4/heparin antibody and who also exhibited a thrombocytopenia or underwent administration of antiplatelet drugs were at significantly increased risk for clinical bleeding.
